# Solution du Probleme de la Population, et de la Subsistence, &c.

**Published:** 1842-10-01

**Authors:** 


SOLUTION DU PROBLEME DE LA POPULATION, ET DE LA SUBSISTENCE, &c. Par *Charles Loudon*, M.D. Octavo, pp. 336. Paris, 1842.

DR. LOUDON (late of Leamington) has long directed his attention to the above interesting investigation, and has now laid his researches and results before the public in the French language. We have no doubt that the political economists, and many of the reading and meditating world, will soon demand a translation of the work into English. The subject does not come strictly within the scope of this Journal, which confines itself almost exclusively to practical medicine and surgery; yet, we do not like to pass it entirely without notice, as the investigation must have occupied the meditations of almost every thinking individual, within or without the profession.

Dr. Loudon observes that it is now generally admitted that there is no limit to the production and re-production of plants and animals—except the excess of their own multiplication, leaving them without subsistence. Mr. Sadler calculated that the product of a single acre of wheat, if sown and re-sown regularly, would, in 14 years, cover the surface of the globe with the same grain! Rules and laws more or less similar apply to fishes and animals. It is calculated that a single herring will give origin to 20,000 of its own species. The same law, though on a limited scale, applies to man himself. We have only to look at China and Ireland, to be convinced that there is no check to population but the want of rice and potatoes. In 1172, when Henry the Second went to Ireland, the population of that island was calculated at 300,000, whereas it is now upwards of eight millions! If the people of the Emerald Isle continue to increase in number at the same rate for 200 years more, the amount will be the enormous number of 128 millions! No man in his senses could, for a moment, believe that the island, fertile though it be, would produce food for such a prodigious mass of human beings. But vice and misery will thin the population whenever it rises up to or beyond the means of subsistence. The doctrine of Malthus and his disciples may be concentrated in the following few words :—"food can only be supplied slowly, and in a definite quantity—procreation goes on rapidly, and is indefinite." The remedy which Malthus proposed, as a preventive check, was the deferring of marriage till the 28th or 30th year, both on the side of the male and the female. Lord Brougham, and many of the most towering intellects in this country, have embraced the doctrines of Malthus. The dread of superabundance of population has so much haunted the minds of men, that it has been proposed to strangle or suffocate a certain portion of new-born infants—and a German writer recommends emasculation!

Dr. Loudon adverts to the violent opposition and personal abuse which Malthus and his disciples have experienced. The doctor candidly acknowledges, that the arguments of Malthus' opponents are destitute of solidity, and calculated only to appeal to the passions of the fanatics and the ignorant.

In the fourth letter Dr. Loudon discusses the causes that check the redundancy of population. These are wars, famines, epidemics, and various other moral and physical agencies. Dr. L. pays a high tribute to the merits of Christianity—a portion of the work which will induce nine-tenths of its readers on the Continent to throw it aside, as the production of a fanatic two centuries behind the level of the illuminati of the present time. To introduce the slightest credence in Christianity in France, is, at once, to cause the work in which that credence is acknowledged, to be thrown into the fire, or torn up in the temples of Cloacina!

We wish we could follow our talented and indefatigable author through his innumerable discursions into all ages, places, and circumstances, rendering his volume one of the most amusing and instructive we have ever perused. But we must confine our notice to a few points connected with physiology and pathology, for obvious reasons.

In the fifth letter Dr. Loudon observes that, as the population of a country, like England, doubles itself in about 25 years, we have no reason to doubt that the whole of the human race sprang from one pair—and that it is not more than five or six thousand years since Adam and Eve commenced their labours in the Garden of Eden.—Credat Judæus! The suckling of children exercises no small influence on population. Females in high life suckle only for a few months—often not at all. If they were as healthy as the middle classes, their families would be enormously large; but their ill health generally prevents them from having many children. Plato and Aristotle fixed the period of marriage at 35 or 37 years—probably because young men were wanted for the wars. Among the Romans and Egyptians marriages generally took place from the age of 40 to 50 years—probably when their military services were over.

In various works recently published, the period of suckling has been recommended to be prolonged to twelve or fifteen months. In the time of Ambrose Paré, the ladies of the French court nursed their infants from 18 to 20 months. The Koran prescribes two years for suckling. Among the Jews the period of suckling was varied; but it appears that ISAAC was at the breast during two years. Dr. Loudon relates a remarkable story of a lady in Warwickshire, who suckled her child to the age of sixteen years! She was told that this conduct was immoral—she therefore weaned her daughter, who quickly died.

Dr. Loudon himself comes to the following conclusion, as one of the first bases of his "SOLUTION OF THE PROBLEM."

> "The period of lactation prescribed by Nature to the human race is *three years;* because it is not till about the end of the third year that the child voluntarily separates itself from the mother's breast."*

The Doctor modifies a little this startling proposition, by stating his opinion that the mother's milk, and that only, should be the sustenance of the infant till it is 14 months old—that is, until it can sit on its seat, and take food in that position, without danger of suffocation. He has seen two instances of children destroyed even by sucking in the horizontal posture. He denies the validity of the physiological dogma that the appearance of the first teeth is the signal for weaning. It might as well be said that, because a child is born with legs it ought to walk from the day of birth. Both the legs and the teeth require time for consolidation before they are fit for their proper office. From the 14th month till the end of the third year, the nourishment should be partly the milk of the mother, and partly cow's or goat's milk, boiled with farinaceous substances. He recommends vegetable food after the third year, till the period of the second dentition, when animal food may be allowed. "This I take to be the true and natural physiology of infancy." Dr. L. endeavours to support this doctrine by comparative anatomy—but not very satisfactorily, as we imagine. If this doctrine were practically adopted, he thinks the period of marriage might be reduced to 18 or 20, instead of 28 or 30 years, as recommended by Malthus. When we consider the number of deaths which take place among children under three years of age, the average period of lactation would not be much above two years. It may be objected that not one female in ten could bear this protracted lactation. We attach no importance to this objection, as we are convinced that mothers suffer more in their constitutions from large families than from long suckling. It is by no means uncommon among the poor Irish women, who have little else than potatoes to live upon, to see children at the breast till they are 18, 20, or 24 months old. The evils, too, of non-lactation or of early weaning, are not few, leaving aside the almost constant state of pregnancy which results from this practice amongst the rich and luxurious. The poor infants themselves suffer still more than their mothers

---

* P. 126.

by this unnatural procedure, which crams their stomachs with the milk of animals, and with various other aliments that are far worse, inducing rickets, scrofula, hydrocephalus, &c. &c.

Our author is very severe on poor Malthus for overlooking protracted lactation, and substituting protracted celibacy. But it is to be remembered that Malthus was neither physician nor physiologist—and that probably it will require both kinds of checks to prevent superabundant population.

Having now disclosed the grand remedy for the evils which threaten this country in particular, engendered in the brain of our highly-gifted friend, Dr. Loudon, and illustrated, if not corroborated, by reference to various nations at various epochs—to physiology—pathology—morality—religion—and last, not least, political economy—we must take leave of our erudite and ingenious author—most strongly recommending his book to readers of all classes, politics, and religions. We believe there is a great deal of truth, and of sound reasoning, in the proposal of our author—and if this be the case, no class of society has it more in their power to propagate the doctrine than our own faculty. It is true that the practical adoption of Dr. Loudon's scheme would be a "heavy blow and great discouragement" to the accoucheurs; but still we have no doubt that they will prefer the good of the community, and especially of their posterity, to their own interests in this world.

It is evident that, in some countries, as Scotland for example, certain moral checks to redundancy of population, in the shape of prudence and foresight, are powerfully operative. In France, too, "they manage these things better," for it is well known that the population does not increase half so fast in that country as in England or Ireland. What the *management* on the other side of the Channel is, this deponent saith not.

